# Psychological dominant stressor modification to an animal model of depression with chronic unpredictable mild stress

**DOI:** 10.14202/vetworld.2023.595-600

**Published:** 2023-03-24

**Authors:** Lisa Pangemanan, Irwanto Irwanto, Margarita M. Maramis

**Affiliations:** 1 Doctoral Program of Medical Science, Faculty of Medicine, Universitas Airlangga, Surabaya, Indonesia; 2 Department of Child Health, Faculty of Medicine, Widya Mandala Catholic University, Surabaya, Indonesia; 3 Department of Child Health, Faculty of Medicine, Universitas Airlangga, Surabaya, Indonesia; 4 Department of Psychiatry, Faculty of Medicine, Universitas Airlangga, Surabaya, Indonesia

**Keywords:** body weight, chronic unpredictable mild stress, modification, psychological, rat, sucrose preference test

## Abstract

**Background and Aim:**

Chronic unpredictable mild stress (CUMS) is a protocol widely used to create an animal model of depression with food deprivation, water deprivation, and physical-dominant stressors as routine procedures. However, human depression mainly involves psychological stressors and does not always involve a lack of food and water; thus, CUMS procedures should be modified accordingly. Therefore, this study aimed to create an animal model of depression, mainly focusing on a psychologically dominant stressor without food and water deprivation.

**Materials and Methods:**

The CUMS and control groups, respectively, received CUMS modification (psychologically dominant stressors without food and water deprivation) for 21 days. A 24-h sucrose preference test (SPT) was used to assess the successful creation of an animal model of depression. Daily food intake measurements, weekly weight monitoring, and weight gain calculations were performed. Either an independent sample t-test or the Mann–Whitney test was used.

**Results:**

Of the 42 rats included, 39 completed the study. Chronic unpredictable mild stress procedures for 21 days significantly reduced the SPT (p < 0.05), mean body weight (p < 0.05), and weekly weight gain (p < 0.05) in the CUMS group compared to the control group. However, the weekly average food intake did not statistically differ between the two groups.

**Conclusion:**

Psychological dominant CUMS modification to an animal model of depression resulted in lower SPT, body weight, and weekly weight gain in the CUMS group than in the control group.

## Introduction

Major depression is an increasing mental problem during adolescence [1–4]. Anhedonia and weight loss without diet, among others, are depression criteria based on the Diagnostic and Statistical Manual of Mental disorders (DSM)-V [3]. An animal depression model is needed to investigate the symptomatology, pathophysiology, and treatment of depression [5, 6]. Chronic unpredictable mild stress (CUMS) is widely used to create a reliable animal depression model [7, 8]. It consisted of repeated, unpredicted, and uncontrollable stressors lasting for weeks [9]. A lower weight gain [8] and anhedonia (decreased sucrose preference test [SPT]) that can be reversed by antidepressant treatment is found in an animal depression model [9–12]. Katz first developed chronic stressors to create an animal depression model using strong stressors such as physical-dominant stressors, psychological stressors, food deprivation, and water deprivation [13]. Willner modified these protocols using a milder stressor still incorporated with food and water deprivation [14]. Water/food deprivation periods can lead to weight loss [15]. Most human stressors are psychological, and the resulting weight loss is not related to diet [16–19]. As various stressors will elicit different manifestations [20, 21], a suitable animal model of depression with stressors mimicking human stressors is needed [22, 23]. Modified CUMS protocols should be designed if a psychologically dominant stressor without food and water deprivation is needed.

This study aimed to develop an animal depression model using psychologically dominant CUMS modification.

## Materials and Methods

### Ethical approval

The protocols were reviewed and approved by the Animal Care and Use Committee, Faculty of Veterinary Medicine, Universitas Airlangga, Surabaya, Indonesia with the number: 2.KE.120.10.2021. All efforts were conducted to ensure the minimal number of animals used and minimize suffering.

### Study period and location

The study was conducted from January to March 2022 in the experimental animal laboratory (LPHC), Faculty of Medicine, Universitas Brawijaya, Malang, Indonesia.

### Animals

A total of 42 male Wistar rats were obtained from PT. Indoanilab (Bogor, Indonesia). The inclusion criteria were male 12-week-old rats, whereas the exclusion criteria were physical illness and disability (assessed by a veterinarian) and death. All animals were group-housed, 2/cage, isolated by a wire mesh separator, and weighed weekly. Subsequently, food, water, and sucrose 1.5% were given *ad libitum* daily every morning, and the leftovers were measured the next day. Fresh water and sucrose solutions were provided daily in separate bottles. A normal 12:12-h light/dark cycle was initiated with lights on at 6:00 am. Laboratory conditions were kept at 23°C ± 2°C with humidity of 40%–70%. After 1 week of the acclimatization period, the animals were randomly assigned to the CUMS and control groups.

### The chronic unpredictable mild stress procedure

**Figure-1:**
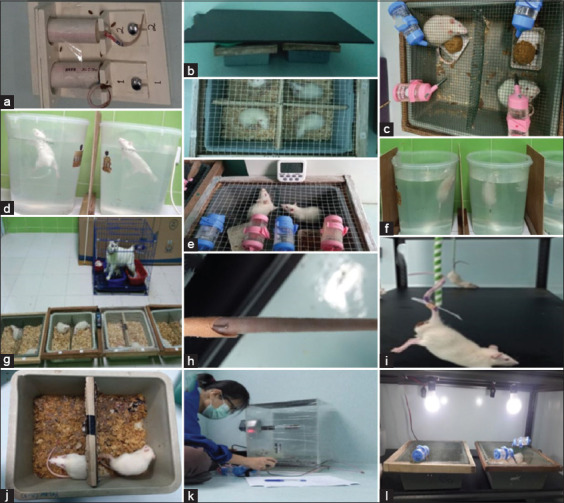
(A) immobilization for 2 h, (b) isolation in a narrow dark space for 4 h, (c) no bedding for 24 h, (d) cold swimming for 3 min, (e) continuous light for 24 h, (f) forced swimming for 5 min, (g) predator exposure for 4 h, (h) tail pierced for 1 h, (i) tail tied for 1 h, (j) wet bedding for 24 h (k) foot shocks for 10 min, and (l) bright light 300–400 lux for 45 min, 2 times.

The CUMS was performed for 21 days based on the previously established protocols (including cold swimming, foot shock, forced swimming, and tail piercing) with psychologically dominant stressor modifications (including immobilization, no bedding, bright light, tail tied, isolation in a narrow dark space, predator exposure, wet bedding, and continuous light) [24–26]. Stressors were randomly initiated once or twice daily. It consists of psychological dominant and physical stressors, as presented in Table-1. The same stressors were not repeated for two consecutive days. Day-by-day protocols are indicated in Table-2, whereas detailed procedures are shown in Figure-1.

**Table-1:** Chronic unpredictable mild stress.

No.	Stressor	Definition	Type
1	Immobilization for 2 h	Rats were individually restrained in a plastic cylinder for 2 h.	Psychological
2	Cold swimming for 3 min	Rats were placed in a cylindrical clear plastic container filled with 4°C water for 3 min.	Physical
3	No bedding for 24 h	Rats were placed in a cage without bedding for 24 h.	Psychological
4	Bright light 300–400 lux for 45 min, 2 times	Rats were given white bright light 300–400 lux for 45 min, 2 times.	Psychological
5	Tail tied for 1 h	Rats’ tail was tied to restrict their movement for 1 h.	Psychological
6	Foot shocks for 10 min	Rats were given 1.5 mA, 35 volt, 4 times for 30 s with 120 s interval foot shock.	Physical
7	Forced swimming for 5 min	Rats were placed in a cylindrical clear plastic container filled with 24°C water for 5 min.	Physical
8	Isolation in a narrow dark space for 4 h	Rats were placed in a narrow dark space for 4 h.	Psychological
9	Predator exposure for 4 h	Rats were exposed to a cat along with a recording of angry cat’s sound (80 dB) for 4 h.	Psychological
10	Tail pierced for 1 h	Rats’ tail was pierced 1cm apart from the base for 1 h.	Physical
11	Wet bedding for 24 h	Rats were placed in a cage with 200 mL water in 100 g sawdust bedding for 24 h.	Psychological
12	Continuous light for 24 h	Rats were given 24 h of light.	Psychological

**Table-2:** Chronic unpredictable mild stress procedure day by day.

Day	Stressor
1	Immobilization for 2 h
2	No bedding for 24 h
3	• Cold swimming for 3 min • Bright light 300–400 lux for 45 min, 2 times
4	• Tail tied for 1 h • Forced swimming for 5 min
5	• Isolation in a narrow dark space for 4 h • Foot shocks for 10 min
6	Predator exposure for 4 h
7	Tail pierced for 1 h
8	Wet bedding for 24 h
9	Continuous light for 24 h
10	Cold swimming for 3 min
11	• Bright light 300–400 lux for 45 min, 2 times • Foot shocks for 10 min
12	• Isolation in a narrow dark space for 4 h • Tail pierced for 1 h
13	• Continuous light for 24 h • Tail tied for 1 h
14	Immobilization for 2 h
15	Predator exposure for 4 h
16	Wet bedding for 24 h
17	No bedding for 24 h
18	Forced swimming for 5 min
19	Cold swimming for 3 min
20	Immobilization for 2 h
21	Tail pierced for 1 h
22	SPT, FST for 15 min (day 1) Bright light 300–400 lux for 45 min, 2 times
23	FST for 5 min (day 2)

SPT=Sucrose preference test, FST=Forced swim test

### Test procedures

The body weight of each rat was measured weekly with an electronic scale. Weekly average meals, weekly weight gain, and SPT were assessed and recorded. The weekly weight gain was defined as the difference in the current minus the prior week. The consumed meal was calculated from the total food given minus the leftovers. The weekly average meal is the mean food consumed by rats in 1 week. An SPT was used to assess the animal depression model and carried out twice, that is, before and 22 days after the CUMS procedure. The preference test was calculated as follows: sucrose percentage (%) = sucrose consumption (mL)/sucrose consumption (mL) + water consumption (mL) × 100%. A forced swimming test was assessed on days 0 and 22; however, due to technical problems, the result cannot be interpreted.

### Statistical analysis

Data were presented as mean ± standard deviation and the difference between variables was analyzed using the independent sample t-test or Mann–Whitney test (non-parametric). p < 0.05 was considered statistically significant. The data analysis was performed using IBM SPSS Statistics software version 23.0 (IBM Corp., Armonk, NY, USA).

## Results

A total of 42 rats were included in this study (33 and nine rats in CUMS and control groups, respectively). Three rats in the CUMS group were excluded (two died during the swimming procedure and another one due to illness). Finally, 39 rats completed the study. The weight of the rats, weekly weight gain, and average meal were normally distributed; thus, t-test was used for statistical analysis. The results indicated that the mean body weight on day 22 (p < 0.01) after CUMS procedures significantly differed from that of the control group, as presented in Table-3. Weekly weight gain between groups showed a significant difference (p < 0.01) in the 1^st^ week and persisted until the 3^rd^ week (Table-4). However, no significant difference (p > 0.05) was observed in the weekly average meals between groups (Table-5). Non-parametric tests were used in non-normally distributed SPT data, whereas T-tests were applied in normally distributed data. The 24-h SPT revealed significant differences between the CUMS (p < 0.01) and control groups (Table-6).

**Table-3:** Mean weight of the rats.

Time	Weight (g) mean ± SD		p-value

	CUMS (n = 30)	Control (n = 9)	
Pre CUMS	168.467 ± 21.591	170.555 ± 14.170	0.787^a^
Week 1 post CUMS	177.800 ± 21.385	192.667 ± 16.658	0.064^a^
Week 2 post CUMS	190.133 ± 22.227	212.889 ± 20.907	0.010*^a^
Week 3 post CUMS	197.333 ± 21.037	226.222 ± 24.030	0.001**^a^

a t-test,

* significant with p < 0.05,

** significant with p < 0.01. CUMS=Chronic unpredictable mild stress, SD=Standard deviation

**Table-4:** Weekly weight gain of the rats.

Time	Weekly weight gain (g) mean ± SD		p-value

	CUMS (n = 30)	Control (n = 9)	
Week 1 post CUMS	9.333 ± 9.076	22.111 ± 7.688	0.000^**^^a^
Week 2 post CUMS	12.333 ± 8.125	20.222 ± 9.615	0.019^*^^a^
Week 3 post CUMS	7.200 ± 7.522	13.333 ± 6.041	0.032^*^^a^

a t-test,

* significant with p < 0.05,

** significant with p < 0.01. CUMS=Chronic unpredictable mild stress, SD=Standard deviation

**Table-5:** Weekly average meal of the rats.

Time	Average meal (g) mean ± SD		p-value

	CUMS (n = 30)	Control (n = 9)	
Week 1	56.015 ± 6.743	59.981 ± 6.309	0.125^a^
Week 2	54.675 ± 8.753	57.299 ± 6.867	0.415^a^
Week 3	53.094 ± 7.873	55.822 ± 7.109	0.358^a^

a t-test. CUMS=Chronic unpredictable mild stress, SD=Standard deviation

**Table-6:** Sucrose preference test of the rats.

Time	Sucrose preference test (%) mean ± SD		p-value

	CUMS (n = 30)	Control (n = 9)	
Pre CUMS	69.627 ± 14.008	68.500 ± 15.230	0.837^a^
Week 1 post CUMS	71.152 ± 13.746	69.908 ± 18.171	0.907^b^
Week 2 post CUMS	75.929 ± 16.405	74.876 ± 25.059	0.604^b^
Week 3 post CUMS	57.526 ± 14.870	82.622 ± 13.107	0.000*^a^

a t-test,

b Mann–Whitney test

* significant with p < 0.01. CUMS=Chronic unpredictable mild stress, SD=Standard deviation

## Discussion

A good animal model should have adequate face, construct, and predictive validity [5]. CUMS is one of the available protocols to create an animal depression model with good validity [5, 9, 27]. It is a well-validated method to model a depressive-like behavior that develops gradually and naturally over time [14], consisting of exposure to various chronic and unpredictable mild stressors [7]. Anhedonia (marked by decreased SPT) is the core symptom of depression found in an animal depression model [5, 12, 28].

Stressors that are commonly found in CUMS procedures include periods of food and water deprivation, shock, cold swim, heat stress, shaker stress, continuous lighting, cage tilt, paired housing, soiled cage, reduced temperature exposure, stroboscopic lighting, novel odors, intermittent white noise (85dB), and presence of foreign objects [14]. Different types of stressors will elicit various responses [29–32]. Multiple and more severe stressors will change the CUMS phenotype [8]. Social stressors are the major of stressors that lead to psychopathology in humans [1, 33]. Since the original CUMS protocols are physically dominant and involve food and water deprivation, modifications to mimic human depression should be performed.

Environmental lighting changes, with the bright light of 300–400 lux for 45 min twice and continuous light for 24 h, will affect mood and result in depression [28, 34–37]. Inescapable foot shock will also create learned helplessness, anhedonia, and despair [7, 38–41]. Foot shock will increase learned helplessness [42, 43]. Restraining stressors such as immobilization for 2 h as well as tail tied for 1 h and isolation in a narrow dark space for 4 h lead to learned helplessness [44, 45]. Predator exposure is a type of psychological stressor that can elicit depressive-like behavior [7, 46]. Cold swimming has also been identified as a cause of learned helplessness [47, 48]. Social isolation is part of CUMS procedures, where the rat is single-housed [8]. Tail pierced, tail tied, foot shock, isolation in narrow dark spaces, and predator exposure were performed based on Maramis *et al*.’s [24] study. Meanwhile, immobilization, no bleeding, bright light, and cold and forced swimming were used in the procedures by He *et al*. [48] with some modifications. Depressive-like behavior can be observed after 21 days of CUMS [49]. In this study, mild stressors with psychological dominant stressors were administered 1–2 stressors daily, with random time to administer for 21 days to make it unpredictable. A total of 12 stressors were chosen to be included in this experiment to obtain the dominant psychological stressor, resulting in learned helplessness and anhedonia. Water and food deprivation was not included as it decreases body weight. Psychologically dominant stressors were carried out to mimic human stressors.

A significant decrease in SPT compared to the control group is a marker of the successful creation of an animal depression model [24, 50]. Additional symptoms of human depression, such as weight/appetite change and psychomotor alteration can be easily assessed in animals [7, 51–53]. Lower weight and reduced weight gain than controls are found in the animal depression model [12, 49, 54]. Weight loss in depression was caused by decreased peripheral leptin [55]. In this study, decreased SPT occurred gradually and became statistically significant after 21 days of CUMS procedures. No significant differences were observed in terms of appetite changes and the amount of food consumed between groups. However, the body weight and weekly weight gain were significantly different between the groups.

This modification protocol can be used if other researchers need an animal depression different with psychologically dominant stressors without food and water deprivation. There are some limitations in this study. First, corticosterone levels were not measured before and after CUMS. Second, neurotransmitter analysis was not performed before and after CUMS. Third, the response to an antidepressant after creating the animal depression model was not analyzed.

## Conclusion

Psychological dominant CUMS modification resulted in an animal depression model with decreased SPT, body weight, and weekly weight gain in the CUMS group compared to the control group. This model resembles psychologically dominant stressors in humans.

## Recommendations

This protocol can be used to create a psychologically dominant animal model of depression. Successful creation of the model can be assessed using the 24-h SPT, body weight, and weekly body weight gain.

## Authors’ Contributions

LP, II, and MMM: Designed the research, methodology, validation, formal analysis, and review and editing. LP: Data collection, and writing original draft preparation. All authors have read, reviewed, and approved the final manuscript.
